# Perinatal Group B Streptococcal Disease Prevention, Minnesota

**DOI:** 10.3201/eid1109.041109

**Published:** 2005-09

**Authors:** Craig A. Morin, Karen White, Anne Schuchat, Richard N. Danila, Ruth Lynfield

**Affiliations:** * Minnesota Department of Health, Minneapolis, Minnesota, USA;; † Centers for Disease Control and Prevention, Atlanta, Georgia, USA

**Keywords:** Group B streptococcus, Perinatal Group B Streptococcal Disease Prevention, dispatch

## Abstract

In 2002, revised guidelines for preventing perinatal group B streptococcal disease were published. In 2002, all Minnesota providers surveyed reported using a prevention policy. Most screen vaginal and rectal specimens at 34–37 weeks of gestation. The use of screening-based methods has increased dramatically since 1998.

Group B streptococci (GBS) emerged as the leading cause of invasive bacterial infections in newborns in the United States in the 1970s. Although the incidence of GBS disease has declined substantially, it remains the leading cause of serious infection in newborns (*1*). Perinatal GBS transmission can be reduced dramatically by diagnosing maternal GBS colonization and administering intrapartum antimicrobial prophylaxis (IAP) during labor and delivery (*2*).

In 1996, the Centers for Disease Control and Prevention (CDC) published consensus guidelines recommending 2 methods of perinatal GBS disease prevention. The screening-based approach recommends obtaining vaginal and rectal cultures at 35–37 weeks of gestation. Women with GBS-positive cultures are offered IAP during labor. The risk-based approach recommends administering IAP to women with GBS risk factors when they go into labor (*3*). These guidelines are believed to have increased use of GBS disease prevention approaches by prenatal care providers, which has led to a decrease in the incidence of GBS disease (*1**,**4*). A 2002 study further indicated that routine screening for GBS would prevent ≈50% more newborn GBS infections than would a risk-based approach (*5*). This study, along with other data, led CDC to publish revised guidelines in August 2002 recommending universal prenatal screening (*6*).

As part of the Minnesota Department of Health Emerging Infections Program, prenatal care providers in Minnesota were surveyed in April 1998 to determine strategies to prevent perinatal GBS disease (*7*). In November 2002, a similar survey was undertaken to determine the extent to which Minnesota providers have adopted the revised 2002 CDC guidelines.

## The Study

In 2002, all licensed obstetricians and certified nurse midwives in Minnesota were surveyed. All family practitioners who listed obstetrics as a secondary specialty and a 20% random sample of the remaining licensed family practitioners were surveyed. In 1998, surveys were mailed to a random sample of 50% of obstetricians and 25% of family practitioners who indicated on their licensure application that they provided prenatal care. All midwives were surveyed. Statistical analysis was performed with EpiInfo software (Centers for Disease Control and Prevention, Atlanta, GA, USA).

Three mailings were sent during each study period. A total of 463 surveys (60% of those mailed) were completed in 2002, and 515 surveys (80% of those mailed) were completed in 1998. Providers who did not provide prenatal care were excluded from further analysis. The final sample included 97 midwives, 189 obstetricians, and 64 family practitioners in 2002 and 102 midwives, 128 obstetricians, and 201 family practitioners in 1998. No significant differences were found in provider characteristics (location, practice type, and number of deliveries performed) from 1998 to 2002.

In 2002, all providers surveyed indicated they had a policy to prevent perinatal GBS disease. Of these, 318 (91% [96% of obstetricians, 92% of midwives, and 73% of family practitioners]) indicated their policy was based upon at least 1 previously published guideline. Family practitioners (p<0.05) and midwives (p<0.05) were significantly more likely to follow published guidelines during 2002 than during 1998.

In 1998, the risk-based approach was the most common method of preventing GBS disease (Table1). In 2002, the screening-based approach was the most common method. In 2002, providers were significantly more likely to have adopted a screening-based approach to prevention than they were in 1998 (p<0.001). In 2002, when risk-based providers were questioned, 14 (52%) of 27 midwives, 5 (50%) of 10 family practitioners, and 6 (32%) of 19 obstetricians indicated they planned to implement the new guidelines.

**Table 1:** Change in policy types to prevent perinatal group B streptococci infection, Minnesota, 1998 and 2002

Policy	Obstetricians		Midwives		Family practitioners	
	1998, n (%) (N = 127)	2002, n (%) (N = 189)	1998, n (%) (N = 104)	2002, n (%) (N = 97)	1998, n (%) (N = 200)	2002, n (%) (N = 64)
Screening-based*	46 (36)	170 (90)	13 (13)	70 (72)	84 (42)	53 (83)
Risk-based*	74 (58)	12 (6)	75 (72)	13 (13)	87 (43)	5 (8)
Risk-based, planning to implement screening-based	–	5 (3)	–	14 (15)	–	5 (8)
Other/unknown†	7 (6)	2 (1)	16 (15)	0	29 (15)	1 (1)

*p<0.001, change from 1998 to 2002 among all prenatal care provider groups. †p<0.005, change from 1998 to 2002 among all prenatal care provider groups.

In 2002, among those who reported a screening-based approach, 262 (89%) of 293 providers routinely collected specimens from both vaginal and rectal sites. Midwives (97%) were more likely than obstetricians (90%) and family practitioners (77%) to collect specimens from both sites. In 2002, midwives (p<0.001) were significantly more likely to use both vaginal and rectal sites to screen for GBS than in 1998. No significant increase was seen in the proportion of obstetricians or family practitioners who screened vaginal and rectal specimens from 1998 to 2002 (Table 2).

**Table 2:** Change in group B streptococci (GBS) screening characteristics among prenatal care providers reporting a screening-based approach, Minnesota, 1998 and 2002

Characteristic	Obstetricians		Midwives		Family practitioners	
	1998, n (%) (N = 45)	2002, n (%) (N = 170)	1998, n (%) (N = 13)	2002, n (%) (N = 70)	1998, n (%) (N = 84)	2002, n (%) (N = 53)
Vaginal/rectal screening	41 (91)	153 (90)	6 (46)	68 (97)*	61 (73)	41 (77)
Screening at 35–37 weeks of gestation	42 (93)	152 (89)	10 (77)	62 (89)	77 (92)	44 (83)
Use selective broth†	12 (27)	122 (72)‡	2 (15)	28 (40)	34 (40)	21 (40)
Penicillin first IAP choice§	35 (78)	138 (81)	7 (54)	60 (86)¶	32 (38)	27 (51)

*p<0.001, use of vaginal/rectal screening from 1998 to 2002. †Prenatal care providers were asked if their laboratory used selective broth to isolate GBS. ‡p<0.001, use of selective broth from 1998 to 2002. §IAP, intrapartum antimicrobial prophylaxis. ¶p<0.01, penicillin as first choice for IAP from 1998 to 2002.

Among providers who used a screening-based approach to prevent perinatal GBS infection in 2002, most (88%) obtained cultures at 35–37 weeks of gestation. No change was seen in the proportion of providers who screened at 35–37 weeks of gestation when responses from the 1998 and 2002 surveys were compared (Table 2).

In 2002, when providers were asked if their laboratories used a selective broth to isolate GBS, 171 (58%) of 293 indicated that they did. Obstetricians were significantly more likely than midwives and family practitioners to report selective broth use in their laboratories. Obstetricians (p<0.001) were significantly more likely to report that their laboratory used selective broth in 2002 than in 1998 (Table 2). Little change was seen among midwives and family practitioners regarding their knowledge of selective broth use from 1998 to 2002.

In 2002, a total of 225 (77%) of 292 providers reported using penicillin most often for IAP. Midwives and obstetricians were more likely than family practitioners to report using penicillin. Midwives (p<0.01) were significantly more likely to use penicillin in 2002 than they were in 1998. Little change was seen in the proportion of family practitioners and obstetricians who used penicillin in 1998 versus 2002 (Table 2).

## Conclusions

The results of this survey suggest that all Minnesota providers have adopted a policy on preventing perinatal GBS disease, and most follow established, published guidelines. In 1998, a risk-based approach to GBS disease prevention was the most common strategy identified by providers. In 2002, screening all pregnant women for GBS was the predominant strategy.

The effectiveness of the screening-based approach depends partly on the sensitivity and specificity of the specimens collected. A previous study by Philipson et al. indicated that swabbing both vaginal and rectal sites significantly increased the sensitivity of isolating GBS compared with swabbing the vagina only (*8*). In our study, 89% of Minnesota providers indicated they routinely collected specimens for GBS screening from both vaginal and rectal sites. Because vaginal and rectal swabs are likely to yield diverse bacteria, selective broth is recommended to limit growth of other organisms, thus increasing the chance of isolating GBS (*9*). In a study by Silver and Struminski, ≈32% of women had false-negative culture results when direct agar plating was used instead of selective broth to isolate GBS (*10*). In our study, most obstetricians (72%) indicated that their laboratories used selective broth; however, less than half of midwives and family practitioners reported using selective broth. Many providers (41%) did not know whether their laboratories used selective broth. A recent survey found that 89% of laboratories that process GBS specimens use selective enrichment broth media for GBS isolation (*11*). Preliminary data from a 2004 survey of laboratories in Minnesota indicated that 92% of laboratories use a selective enrichment broth media to isolate GBS (Minnesota Department of Health, unpub. data).

Collecting cultures late in the gestational period is more likely to detect women who are colonized when they deliver, compared to screening at an earlier stage of a woman's pregnancy. In 2002, most (88%) providers who reported a screening-based approach to perinatal GBS disease prevention obtained cultures at 35–37 weeks of gestation.

Research in the 1980s showed that administering antimicrobial prophylaxis to women who are colonized with GBS was effective in preventing disease in newborns. Because of its narrow spectrum, penicillin remains the preferred drug of choice. Ampicillin, a broader-spectrum agent, is considered an acceptable alternative. In our study, >80% of obstetricians and midwives reported using penicillin as their first choice for IAP. Although family practitioners were significantly more likely to use penicillin in 2002 than in 1998, only 51% of family practitioners listed penicillin as their first choice.

Several factors should be considered when interpreting the results of this study. First, the survey was conducted only among Minnesota providers, so the results may not be generalized to other states. Second, the overall response rate was 80% in 1998 and 60% in 2002. This decrease is most likely explained by a sampling change in which a greater proportion of family practitioners with a history of providing prenatal care were sampled in 1998 than in 2002. We suspect that most family practitioners who failed to complete the survey in 2002 did so because they did not provide prenatal care. When characteristics of responders in 1998 and 2002 were compared, no significant differences were noted regarding location of practice, practice type, size of practice, and median number of deliveries performed. Finally, surveys are measures of reported practices and may not reflect actual services provided.

Prenatal care providers, especially family practitioners, should continue to discuss and establish policies regarding perinatal GBS disease prevention. Providers should be educated about optimal specimen sites and timing of screening. Education on using selective broth medium to isolate GBS should be provided to clinicians and laboratories. In addition, clinicians should be familiar with the appropriate antimicrobial agents used for IAP and ensure rapid drug administration when it is indicated.
